# Ethical Issues for Direct-to-Consumer Digital Psychotherapy Apps: Addressing Accountability, Data Protection, and Consent

**DOI:** 10.2196/mental.9423

**Published:** 2018-04-23

**Authors:** Nicole Martinez-Martin, Karola Kreitmair

**Affiliations:** ^1^ Stanford Center for Biomedical Ethics Stanford, CA United States

**Keywords:** ethics, ethical issues, mental health, technology, telemedicine, mHealth, psychotherapy

## Abstract

This paper focuses on the ethical challenges presented by direct-to-consumer (DTC) digital psychotherapy services that do not involve oversight by a professional mental health provider. DTC digital psychotherapy services can potentially assist in improving access to mental health care for the many people who would otherwise not have the resources or ability to connect with a therapist. However, the lack of adequate regulation in this area exacerbates concerns over how safety, privacy, accountability, and other ethical obligations to protect an individual in therapy are addressed within these services. In the traditional therapeutic relationship, there are ethical obligations that serve to protect the interests of the client and provide warnings. In contrast, in a DTC therapy app, there are no clear lines of accountability or associated ethical obligations to protect the user seeking mental health services. The types of DTC services that present ethical challenges include apps that use a digital platform to connect users to minimally trained nonprofessional counselors, as well as services that provide counseling steered by artificial intelligence and conversational agents. There is a need for adequate oversight of DTC nonprofessional psychotherapy services and additional empirical research to inform policy that will provide protection to the consumer.

## Introduction

Given the pressing need for expanding mental health care options [1], there is an understandable enthusiasm for applying digital technology—including mobile phones, apps, and wearables—for improving mental health care and making therapy more accessible. Mobile phones are owned by at least 80% of the Americans [2], and digital technology allows for innovations in delivering therapy to users wherever they are located. The number of mental health therapy apps is rapidly proliferating, and many do not involve mental health professionals, or even humans, in providing talk therapy for consumers. However, many of the ethical obligations concerning mental health care are rooted in the traditional therapist-patient relationship [3]. But what becomes of professional ethical obligations when the therapist is an algorithm? Attention needs to be paid to the ways that disrupting mental health care can also disrupt the relationships and obligations meant to provide a foundation of trust, transparency, and safety for mental health care.

This paper focuses on the ethical challenges presented by direct-to-consumer (DTC) digital psychotherapy that does not involve oversight by a mental health professional. Previous scholarship has raised ethical concerns regarding the broader landscape of digital mental health technology, identifying safety, transparency, and privacy as key challenges [4-8]. However, questions regarding appropriate oversight and what ethical duties are owed to consumers, and by whom, present particular ethical challenges for DTC digital psychotherapy services. We seek to outline the ethical challenges regarding accountability, safety, informed consent, and protection of consumer data in order to provide a foundation for development of appropriate guidelines and practices that support innovation in DTC digital mental health care while protecting against ethical risks.

The issues raised in this paper are, for the most part, specific to the US regulatory context. It should be noted, however, that the European Union (EU) provides significantly more protection for personal data and informed consent, as well as more stringent regulation of health apps and devices, and thus most of the concerns raised here would not apply to the EU [9,10]. In the United States, there is currently minimal regulation [4,11] of digital mental health technology. Some digital talk therapy apps may be classified as a “medical device” and thus subject to regulation by the Food and Drug Administration (FDA) [10-12]. The FDA has announced new initiatives to regulate digital health technology; however, it is not yet clear the extent to which, and how, the FDA will opt to regulate DTC digital psychotherapy apps [13]. Furthermore, while safety and effectiveness are important issues, increased FDA regulation of DTC psychotherapy would not address other important ethical challenges such as privacy and informed consent.

The American Psychological Association has made an effort to provide guidance to their members regarding appropriate standards for the use of digital technology in mental health practice [7,14]. However, this guidance is aimed more at mental health professionals, and it is not clear that consumers will rely upon these avenues of guidance. Additionally, while some states and professional organizations may have the resources to address specific DTC services that can be said to be practicing mental health therapy without a license, there is a larger policy question that needs to be engaged regarding which professional ethical obligations will need to be applied to this growing domain from which people can receive mental health care. Certain ethical challenges, such as privacy, will need to be addressed with effective policy and guidelines for protecting the consumers of DTC digital psychotherapy.

## Types of Direct-to-Consumer Digital Psychotherapy

The types of digital mental health psychotherapy examined in this paper are DTC digital psychotherapy services that are not mediated or overseen by a professional mental health provider, such as psychiatrist or licensed mental health therapist, and for which the ethical obligations of a professional therapist therefore do not apply. There are a variety of approaches being used to deliver DTC psychotherapy with digital technology. The services examined here include (1) digital technology services that connect the user to talk therapy (via text or voice) provided by a person with minimal to no training in mental health services or (2) services that provide an interactive software platform (eg, chatbot, conversational agent) to provide psychotherapy. Digital mental health services that utilize individuals with minimal or no mental health training as therapist-substitutes could be considered simply analogous to peer counselors. However, we include these types of “peer counseling” services in our discussion of ethical challenges because, when moved to a digital platform and conducted without any professional oversight, there needs to be a better understanding of how to ensure that the potential benefits of these services are sufficient to outweigh potential risks to users, particularly when it comes to privacy and safety.

## Protecting User Data

Privacy is a major concern when it comes to protecting the interests of users of DTC digital psychotherapy. Psychotherapy involves the sharing of deeply personal and sensitive information by a patient [15]. With DTC digital psychotherapy, behavioral health information is shared, stored, and potentially sold to third parties in the consumer domain, outside of HIPAA or the health care institutions that traditionally protect health information [16,17]. Business models for services that provide low-cost or free digital psychotherapy may share or sell user data for marketing or other purposes for which the user may not understand or be able to foresee. Furthermore, digital psychotherapy apps available through employer wellness programs can leave users vulnerable to invasion of privacy and employment discrimination [18]. Although it is sometimes argued that the Internet has made people less concerned about protecting their privacy, users may feel greater concerns when it comes to the uses of sensitive behavioral data. Recently, Facebook was revealed to have allowed marketers to target anxious and depressed teenagers with ads [19], and Google was exposed for providing a platform for referrals to substandard substance abuse centers [20]. These incidents highlight how digital behavioral data can be shared and sold for practices that put the consumer at risk.

Consumers may not be aware of the various ways the service may collect and analyze their data. An app may not be collecting just the talk therapy chats but even location and other data. Therapy apps can potentially utilize computational behavioral analyses and machine learning to analyze user information such as voice inflection, location data, or screen taps, collected passively or actively through apps or wearables, in order to assess and predict cognitive and behavioral states [21,22]. For example, changes in voice can be used to predict risk of psychotic episodes [23]. Thus, new kinds of behavioral risk data may be created, making it difficult to foresee the potential repercussions for consumers regarding the data being shared. There will need to be further research to support guidelines for how these kinds of data from behavioral analytics should be protected and used. For example, if an algorithm determines that a user presents a threat of imminent harm to themselves or another [24,25], should there be an accompanying duty to report that information to someone? At a minimum, information for these services should make it clear to users who will have access to the data, what kind of data is being collected, and how it is being used and stored. However, even with transparency, there needs to be attention at a policy level regarding legitimate uses of consumer behavioral data. For an example of stronger data protections, the EU will soon be implementing General Data Protection Regulation (GDPR). The GDPR expands protection for personal data in the digital domain, including giving consumers easier access to their own data and requiring that companies explain how data will be used in a clear, understandable terms [26].

Confidentiality involves the obligations of providers regarding keeping data private. Confidentiality is considered necessary for effective therapy in order to allow clients an environment to share personal information for therapeutic purposes. Nonprofessional DTC digital psychotherapy providers do not have a duty to keep consumer data confidential and generally state so in the terms and conditions. For example, 7 Cups of Tea, a service that uses minimally trained peer listeners to provide talk therapy, advises clients that “chats or transcripts, being captured in any format, [can be] controlled, processed and shared by 7 Cups of Tea with third parties as designated solely by 7 Cups of Tea” [27]. However, consumers may not adequately read through dense terms and conditions and thus may be unaware of the fact that their digital psychotherapy service does not keep data confidential. Consumers may also be unaware that, with many mental health apps, their data could be accessed by subpoena and/or for legal proceedings [28]. Furthermore, concerns over lack of confidentiality could impede consumer uptake of otherwise safe and effective DTC digital psychotherapy services. It will be important to consider what the proper limits for confidentiality might be, such as how DTC digital psychotherapy services should approach a duty to warn. At a policy level, there is a need for stakeholders, from industry, developers, mental health consumers, clinicians and activists, to contribute to establishing regulations and industry guidelines to protect consumer privacy and establish a foundation of trust for DTC digital therapeutic interactions.

## Safety

Evaluating safety and effectiveness has been identified as a key challenge for digital health technology in general, given that only a small proportion of the thousands of mental health apps are backed by evidence-based studies [29]. For DTC digital psychotherapy specifically, in order to be ethically justified, the services must deliver sufficient benefits to balance against any risks to the consumers. Substandard therapy provided by DTC digital psychotherapy can cause direct harms through incorrect advice, or, it could divert people from reaching appropriate and more effective mental health services [30]. Empirical research into how consumers engage with DTC digital psychotherapy in real-world conditions is needed to help inform the types of oversight or standards that can help ensure that the benefits of DTC digital psychotherapy outweigh potential risks and that risks are minimized.

The “commercialization gap” in digital health technology means that apps developed by clinical researchers are subjected to more rigorous testing for safety and effectiveness, while private sector products are more likely to be designed to maximize user engagement [31]. That can lead to a situation where less effective DTC digital psychotherapy apps end up being more engaging and popular with consumers. Consumer apps contain features that have a primary goal of getting people to use the app as often as possible. Features that activate the reward systems in the brain can be useful to keep people engaged in therapy but also may have downsides, such as addictive behavior and anxiety, which would be in conflict with therapeutic goals [32-35]. The potential for conflict between the design features of the technology and the goals and effectiveness of treatment need to be better understood [36,37]. This is an area where encouraging collaboration between industry and clinical researchers can be useful to find an appropriate balance for development of digital technology that is safe and effective while also sufficiently engaging. Such collaboration can also improve how values are prioritized and incorporated into the technology design, such as utilizing design to provide ways to give users more control over and benefit from their data. At the same time, given the rush for investment and general high enthusiasm for digital mental health technology [38], attention is needed to ensure that the incentives to produce a winning app do not override meticulous empirical research standards.

Current research indicates that digital psychotherapy that involves professional oversight is safer and more effective than DTC services [39,40]. DTC digital therapy services seem to be most appropriately targeted at users with comparatively mild or straightforward mental health issues—and indeed many services generally include a statement in terms and conditions that consumers with severe mental disorders should seek assistance elsewhere [41]. However, empirical research is still needed to inform industry standards and best practices regarding design features, business models, and best practices for safe and effective DTC digital psychotherapy use for specific populations. For example, 7 Cups of Tea, in its user information, advises people with severe mental illness to go elsewhere, but, in its main interface, bipolar disorder, eating disorders and cutting are included in the list of issues that nonprofessional “listeners” can address for clients [27], despite these being complicated conditions that can present serious risks. Evidence-based standards for the safe use of these DTC services by adolescents are also needed, because of potential differences in patterns of usage and expectations, as well as differences in the impact of these interventions on that population [42-44]. Nonprofessional mental health care that may be effective in face-to-face circumstances, such as peer counseling [45,46], requires sufficient research to ascertain best practices in the digital realm. Some digital services encourage users as young as 13 years old and allow “peer counselors” as young as 15 years [47] to provide advice on issues that can range in severity. Such practices highlight the importance of developing evidence-based guidelines for how adolescents use these services.

## Accountability and Liability

The professional codes of ethics guiding professional mental health therapists and clinicians contain duties to maintain confidentiality, provide competent care, a duty to protect, and generally safeguard the interests of the client [48-50]. When there are questions of malpractice or liability, professional therapy codes are meant to provide avenues for patients to report the therapist, identify duties breached, and outline standards for evaluating that liability. A primary challenge regarding DTC digital psychotherapy is that these types of obligations do not clearly apply to DTC providers. DTC digital therapy services hold themselves out as a reasonable alternative to therapy, yet there is little accountability for chatbots, untrained peers, and algorithms for entering into a safe and trusting therapeutic relationship with clients [15,51].

Product liability laws could provide an avenue to address certain harms. However, many DTC digital psychotherapy services effectively limit liability with statements in the terms and conditions that they are not providing professional therapy and that minimize any responsibilities to the consumer. For example, 7 Cups of Tea, “makes no representation or warranty” as to the accuracy of advice or abilities of the listeners on its service [27]. While this may provide legal protections for the company, there is a gap in accountability for providing a foundation of trust and safety for consumers with mental health needs seeking therapy. On a policy level, there needs to be consideration of whether there are certain ethical obligations that will need to apply to DTC digital psychotherapy.

## Informed Consent

Informed consent requires that patients have a clear understanding of the risks and benefits, available alternatives, and relevant facts pertaining to a therapy [52]. For DTC digital psychotherapy services, statements advising consumers of potential risks or how data are handled are generally provided through the terms and conditions section or user agreement. The set of disclaimers found in these services’ user agreements are generally presented in dense and formal language that many people find difficult to parse. Most people do not spend the time to carefully read through terms and conditions language on websites or apps [53,54]. Because the DTC digital therapy services present themselves as a kind of therapy, consumers may assume that their interactions with the service involve the kind of ethical obligations that are a part of professional therapy, making it particularly important to ensure that users understand that those obligations do not apply. Considering that potential users of these services are people with mental health issues, including adolescents, more attention needs to be paid to ensure that consumers are presented with an appropriate overview of risks and benefits before using the service. Basic measures to improve user agreements include making sure that the reading level of the user agreement is not too high [55,56]. There are also innovations available for improving informed consent on digital technology platforms, such as slowing down the consent process with interactive screens, having bullet-point summaries of the most important risks or warnings, or providing video/audio content to clarify risks and benefits [57,58]. Furthermore, note that in the EU, the GDPR does not allow for general broad consent to unspecified uses of the consumer’s data as they arise, although it is less strict when it comes to use of data for scientific research [59]. Such protections in the United States would protect consumers from practices that bury key information, such as how personal data will be stored, shared, and used, in the terms and conditions.

## Conclusions

The use of digital technology for mental health care is exciting and, in addition to improving treatment, could make mental health care less expensive and easier to access for many people with mental health problems. Addressing the ethical challenges presented by DTC digital psychotherapy may also help to encourage consumers to take advantage of safe and convenient digital mental health resources. There is a need for stakeholders, from software developers, health care, and consumers, to be involved in the creation of standards and best practices that adequately address the ethical challenges raised in this paper. Additionally, appropriate regulatory oversight, particularly when it comes to safety and the protection of consumer’s behavioral data, will be critical. Further empirical research into DTC digital psychotherapy practices is needed to inform appropriate policy and best practices. For certain services, there also may be a need to improve the authentication of users, in order to reduce potential risks from children or other vulnerable groups using inappropriate DTC digital therapy. It will also be important to encourage collaboration between industry and developers, mental health consumers, clinicians, and ethicists in order to consider how values may be prioritized in design, such as improvements in informed consent or considering how programming interfaces may be used to give users more control over their data.

## Abbreviations

DTC: direct-to-consumer
EU: European Union
FDA: Food and Drug Administration
GDPR: General Data Protection Regulation
